# Rapid decline in the susceptibility of *Plasmodium falciparum* to dihydroartemisinin–piperaquine in the south of Vietnam

**DOI:** 10.1186/s12936-017-1680-8

**Published:** 2017-01-13

**Authors:** Ngo Viet Thanh, Nguyen Thuy-Nhien, Nguyen Thi Kim Tuyen, Nguyen Thanh Tong, Nguyen Thuy Nha-Ca, Le Thanh Dong, Huynh Hong Quang, Jeremy Farrar, Guy Thwaites, Nicholas J. White, Marcel Wolbers, Tran Tinh Hien

**Affiliations:** 1 Oxford University Clinical Research Unit, Wellcome Trust Major Overseas Programme, 764 Vo Van Kiet Street, Ward 1, District 5, Ho Chi Minh City, Vietnam; 2 Institute of Malariology, Parasitology, and Entomology, Ho Chi Minh City, Vietnam; 3 Institute of Malariology, Parasitology, and Entomology, Qui Nhon, Vietnam; 4 Nuffield Department of Medicine, Centre for Tropical Medicine and Global Health, University of Oxford, Oxford, UK; 5 Mahidol Oxford Tropical Medicine Research Unit, Mahidol University, Bangkok, Thailand

**Keywords:** Artemisinin resistance, Piperaquine resistance, Parasite clearance half-life

## Abstract

**Background:**

Artemisinin resistant *Plasmodium falciparum* has emerged in the countries of the Greater Mekong sub-region posing a serious threat to global malaria elimination efforts. The relationship of artemisinin resistance to treatment failure has been unclear.

**Methods:**

In annual studies conducted in three malaria endemic provinces in the south of Vietnam (Binh Phuoc, Ninh Thuan and Gia Lai) between 2011 and 2015, 489 patients with uncomplicated *P. falciparum* malaria were enrolled in detailed clinical, parasitological and molecular therapeutic response assessments with 42 days follow up. Patients received the national recommended first-line treatment dihydroartemisinin-piperaquine for three days.

**Results:**

Over the 5 years the proportion of patients with detectable parasitaemia on day 3 rose steadily from 38 to 57% (P < 0.001). In Binh Phuoc province, the parasite clearance half-life increased from 3.75 h in 2011 to 6.60 h in 2015 (P < 0.001), while treatment failures rose from 0% in 2012 and 2013, to 7% in 2014 and 26% in 2015 (P < 0.001). Recrudescence was associated with in vitro evidence of artemisinin and piperaquine resistance. In the treatment failures cases of 2015, all 14 parasite isolates carried the C580Y *Pf*kelch 13 gene, marker of artemisinin resistance and 93% (13/14) of them carried exoE415G mutations, markers of piperaquine resistance.

**Conclusions:**

In the south of Vietnam recent emergence of piperaquine resistant *P. falciparum* strains has accelerated the reduced response to artemisinin and has led to treatment failure rates of up to 26% to dihydroartemisinin-piperaquine, Vietnam’s current first-line ACT. Alternative treatments are urgently needed.

**Electronic supplementary material:**

The online version of this article (doi:10.1186/s12936-017-1680-8) contains supplementary material, which is available to authorized users.

## Background

Over the past 30 years there have been substantial reductions in malaria mortality and morbidity across the Greater Mekong sub-region. This has been driven largely by increased deployment of artemisinin-based combination therapy (ACT) for falciparum malaria which have replaced the monotherapies which successively fell to resistance. In Vietnam, the number of clinical cases of malaria decreased from 1,672,000 with 4650 deaths in 1991 to 19,252 with 3 deaths in 2015 [1]. Dihydroartemisinin-piperaquine (DP) was adopted in 2005 as first-line treatment and is the most extensively used anti-malarial drug in Vietnam. The success of the National Malaria Control Programme (NMCP) led to the initiation of “Malaria Elimination by the year of 2025,” a project endorsed with strong political commitment. Neighbouring countries have also set malaria elimination targets for the near future. These laudable objectives are now threatened by the emergence and spread of artemisinin resistant *Plasmodium falciparum*. First recognized in Western Cambodia 10 years ago, artemisinin resistant malaria parasites are now prevalent across the Greater Mekong subregion from the Myanmar-India border in the west to the coast of Vietnam in the East [2, 3]. To characterize the extent of artemisinin resistance and its effects on therapeutic responses to ACT, the Global Plan for Artemisinin Resistance Containment [4] recommended regular in vivo and in vitro assessments. This study reports on the results of sequential studies conducted in the southern provinces Binh Phuoc, Ninh Thuan, and Gia Lai of Vietnam.

## Methods

### Study sites

**Fig. 1:**
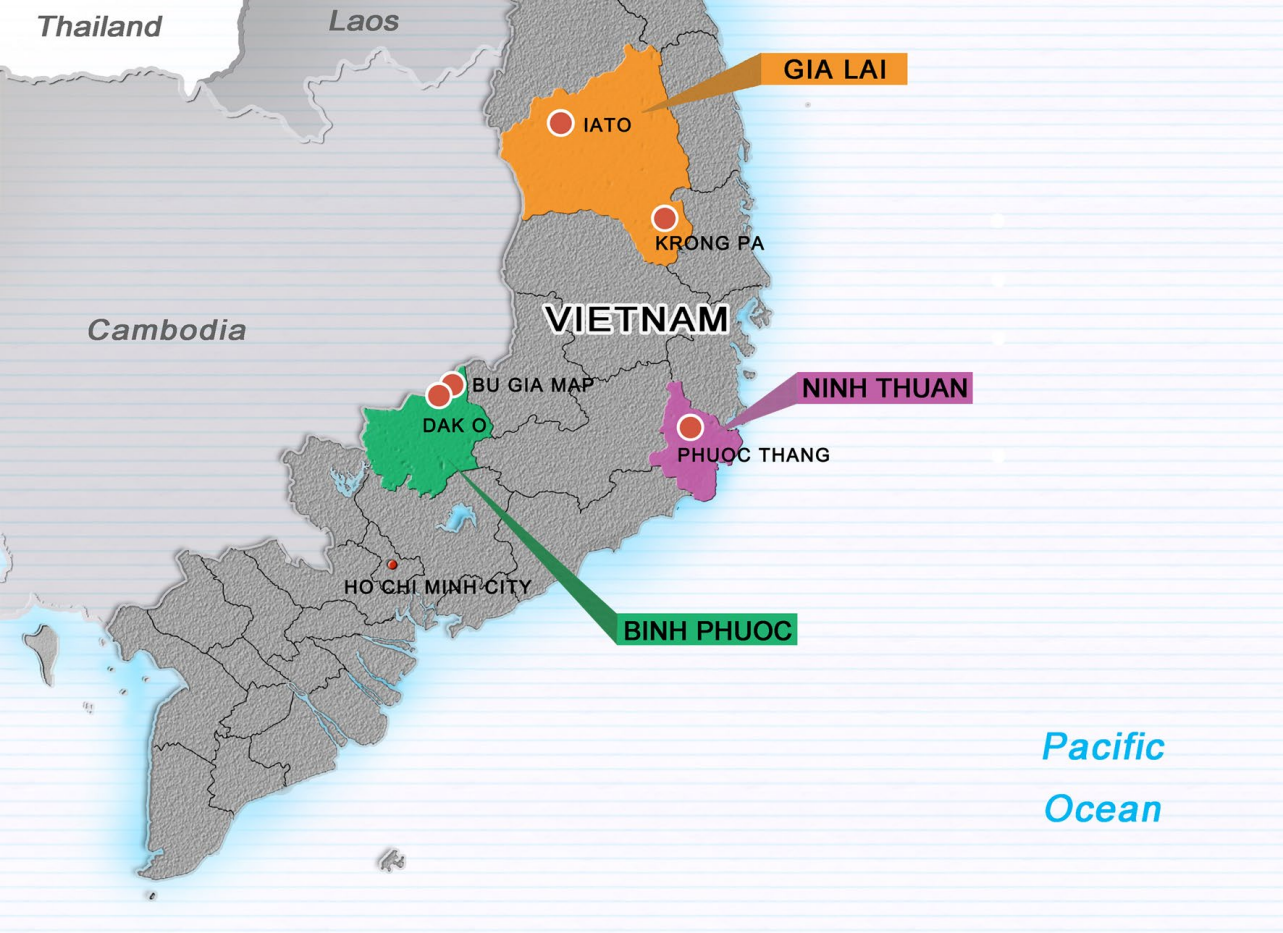
The study sites in Vietnam

The study was conducted from August 2011 to December 2015 in (i) the Bu Gia Map and Dak O communes located in the Bu Gia Map district, Binh Phuoc province, 130 km northeast of Ho Chi Minh City and 100 km west of the Cambodian border (In 1989, Binh Phuoc was the first province in Vietnam to use artemisinin monotherapy); (ii) the IaTo commune, Ia Grai district, and then the Krong Pa Medical Center (this study site replaced the laTo site in 2012 because of slow recruitment), both are in Gia Lai province in the southern highlands; (iii) the Phuoc Thang commune, Bac Ai district, Ninh Thuan province in central Vietnam (2013–present) (Fig. 1).

### Recruitment of study participants

Inclusion and exclusion criteria followed the World Health Organization’s protocol for surveillance of the therapeutic efficacy of anti-malarial medicines [5]. Patients presenting with uncomplicated *P. falciparum* malaria detected using microscopy, aged three years and above with an asexual parasitaemia between 10,000- and 200,000 parasites/µL were invited to participate in the surveillance program. Participants aged 15 years and above provided written informed consent. Parents or guardians granted informed consent on behalf of their children. A minimum sample of 50 patients was required for the study to be representative (n ≥ 50) [5]. We therefore planned to enroll 50–75 subjects per site per year.

### Treatment of malaria and follow-up

Patients with uncomplicated malaria who met the study inclusion criteria were enrolled, screened, and treated on-site daily with dihydroartemisinin-piperaquine (DP) (Arterakin, CV Artecan) for 3 days, following the National Malaria Treatment Guidelines [6]. DP tablets containing 40 mg of dihydroartemisinin and 320 mg of piperaquine phosphate were obtained from OPC Pharmarceutical Joint-Stock Company (Ho Chi Minh, Vietnam) and distributed by the NMCP. The number of DP tablets administered at 0, 8, 24, and 48 h was administered according to body weight (Additional file 1). All doses were administered and recorded under the supervision of a qualified member of the staff designated by the principal investigator. The subjects were observed for 30 min for adverse reactions or for vomiting after taking the medication. Any patient who vomited during this period was re-treated with the same dose and observed for a further 30 min. If vomiting occurred between 30 and 60 min after administration, half the dose was repeated. If the patient vomited again, he or she was withdrawn and offered rescue therapy. Patients were monitored for 42 days. The follow-up comprised a fixed schedule of examinations on days 3, 7, 14, 21, 28, 35, 42 and included clinical and laboratory testing. PCR genotyping was used to distinguish between recrudescence and reinfection [7].

## Clinical and laboratory procedures

### Microscopy examination of blood

Thick and thin blood films were examined at screening (day 0) then every 6 h until two consecutive slides were negative as well as on each day of scheduled follow up or on any other day if the patient returned and parasitological reassessment required. Specimens were labeled to maintain anonymity (screening or study number, day of follow-up, and date). Thick and thin blood smears were examined were stained with 10% Giemsa in pH 7.2 buffer solution for 15 min. Parasitaemia was estimated by counting the number of asexual parasites per 500 leucocytes in the thick blood film or per 1000 red blood cells on a thin film then calculated followed WHO guideline. All slides were read by two qualified microscopists independently, and the average of the two counts was taken as the parasite density. Blood smears with discordant results (differences in assessments of species, parasite density >50%, or the presence of parasites) were re-examined by a third independent microscopist, and parasite densities were calculated by averaging the two closest counts [5].

### Genotyping of malaria parasites

To minimize discomfort from repeated finger pricks, four drops of blood were collected on Whatman 3MM filter paper (Whatman, UK) during screening or enrolment, and each time a blood smear or blood draw sample was required according to the protocol on day 7 and after. Specimens were anonymized and stored in individual plastic bags containing desiccant pouches that were protected from light, humidity, and extreme temperatures. If treatment failed, paired filter paper samples were used for parasite DNA extraction and genotyping based on polymorphisms in the genes encoding the merozoite surface proteins MSP-2 and MSP-1 as well as GLURP follow standard procedures [7].

### K13 single-nucleotide polymorphism (SNPs) genotyping

Taqman SNP genotyping was used to detect mutations in the K13-propeller gene at the field sites within 1 or 2 days after collection. The total genomic DNA from the dried blood spot samples were extracted by a MagNa Pure 96 Instrument automated extraction system using MagNa Pure 96 DNA and Viral NA Small Volume Kit (Roche, Switzerland). Seven positions on K13-propeller gene (Y493H, R539T, I543T, P553L, C580Y, V568G and P574L) were chosen to genotyping based on result of previous reports on K13 in Vietnam parasite population [8, 9]. Ten ng of DNA template was genotyped at 7 positions described above separately in 10 μL volume reactions including 0.5 μL Taqman primer probe mix (Applied Biosystems), 5 μL Light Cycler Probe master mix (Roche, Switzerland). The cycling conditions were 10 min at 95 °C followed by 50 cycles of 15 s at 95 °C, 1 min at 60 °C and hold at 37 °C for 15 min. Allelic discrimination analysis was performed to determine the genotype of the samples. All samples with mutations and 30 samples with no mutations at 7 SNPs described above (randomly chosen) were retested by capillary sequencing to confirm the results of SNP genotyping.

### In-vitro susceptibility assays

Ex vivo responses to dihydroartemisinin (DHA) and in vitro reponses to piperaquine were assessed in 9/14 *P. falciparum* samples of recrudescence cases (collected from patients involved in the DP efficacy monitoring study in 2015–2016 in Binh Phuoc Province) by the ring stage survival assay (RSA) [10] and piperaquine survival assay (PSA) [11], respectively. The results were expressed as the survival rate of parasites after 6 h drug exposure for RSA and 48 h for PSA. The DHA and piperaquine sensitive 3D7 and K1 *P. falciparum* lines were used as controls in the RSA and PSA.

### Piperaquine resistant marker testing

DNA samples from recrudescence cases and ACPR cases were sent to Welcome Trust Sanger Institute for genotyping to detect mutation at position 415 in putative exonuclease gene (exoE415G) followed a protocol described recently [12]. Copy number of plasmepsin 2 encoding gene were detected by real time qPCR followed a procedure shared by the same group [12].

### Outcome measures

The primary objective of this study was to monitor for artemisinin resistance by assessing parasite clearance times (PCT100), specifically, the proportion of patients with clearance times >72 h after starting DP treatment [13]. The PCT100 was defined as number of hours from the first treatment dose to the first of two consecutive negative thick blood films for asexual *P. falciparum* parasites in 500 fields. Secondary outcomes were as follows: (i) The parasite clearance half-life (PC_1/2_) was defined as the time for parasitaemia to decrease by 50% during the log-linear phase of parasite clearance as calculated by the WorldWide Antimalarial Resistance Network parasite clearance estimator [13]. (ii) The fever clearance time (FCT) was defined as the number of hours from the first treatment dose to the start of the first sustained 24 h period without fever (<37.5 °C). (iii) Treatment failure was defined according to WHO guidelines [5] [proportion of early treatment failures (ETFs), late clinical failures (LCFs), late parasitological failures (LPFs), and adequate clinical and parasitological responses (ACPRs) by day 42]. PCR-uncorrected and PCR-corrected failure rates were reported. Recrudescence was defined as detection of identical alleles for each of the three polymorphic markers in the pretreatment and post-treatment samples. Infections in which one or more alleles differed were classified as new infections [7]. (iv) The relationship between treatment outcomes and the prevalence of the K13-propeller mutations was characterized.

### Statistical analysis

The proportion of subjects with a PCT >72 h was calculated using the Kaplan–Meier method. The risk of treatment failure was estimated using the Kaplan–Meier estimator (intention-to-treat analysis) and as a proportion of evaluable patients only (per protocol analysis). At each site, changes in outcomes were assessed using linear trend tests. Specifically, the outcome was regressed according to calendar time using the Cox proportional hazards model (parasite clearance time, fever clearance time, and intention-to-treat analysis of the time to treatment failure), linear regression (parasite clearance half-life), or logistic regression (per protocol analysis of treatment failures and prevalence of the K13-propeller mutation). All analyses were performed using R v.3.3.1 software (R Foundation for Statistical Computing, Vienna, Austria).

### Ethics

The protocol, informed consent documents, relevant supporting information, and all patient recruitment information were approved by the Ethics Committees of the Ho Chi Minh and Qui Nhon Institutes of Malaria, Parasitology and Entomology and the Oxford University Tropical Research Ethics Committee. Any person who decided not to participate in the study was examined, treated, and followed by health facility staff according to the standard of care recommended by the Ministry of Health [6].

## Results

**Fig. 2:**
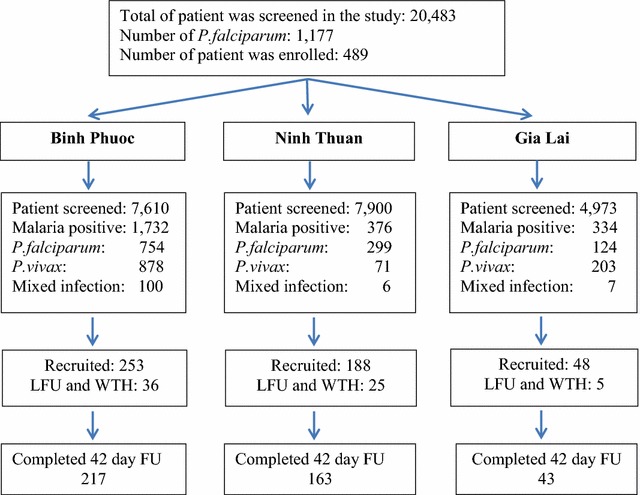
Study flow diagram

**Fig. 3:**
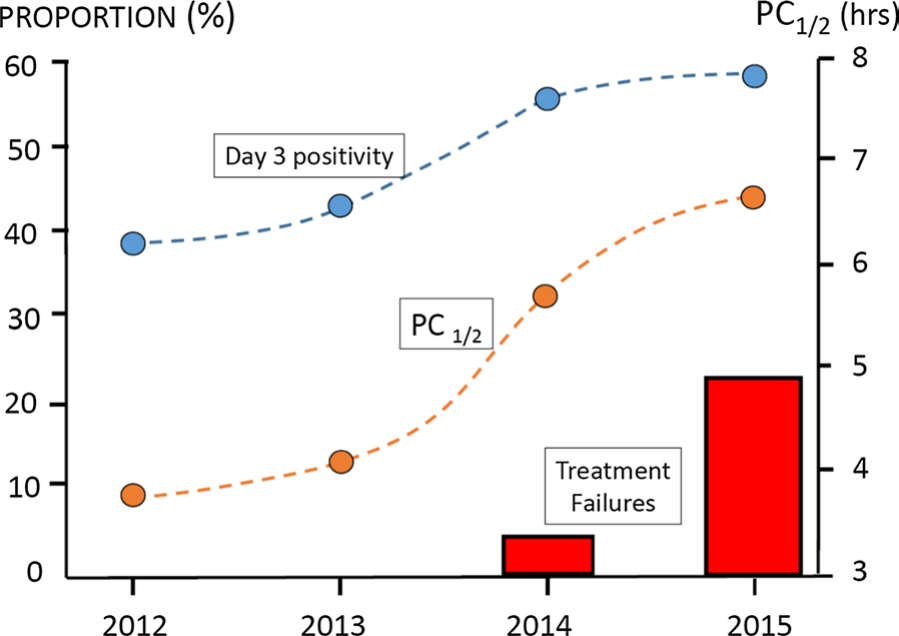
Proportion of patients with positive smear on day 3, parasite clearance half-life (PC_1/2_) and the treatment failure rate following dihydroartemisin–piperaquine treatment in Binh Phuoc from 2012–2015 in Vietnam

Between August 2011 and December 2015, 489 patients with uncomplicated *P. falciparum* malaria were recruited: 253 in Binh Phuoc; 188 in Ninh Thuan; and 48 in Gia Lai. The 42-day follow-up schedule was completed by 423 (87%) subjects (Fig. 2). Adults (>15 years of age) represented 77% (375/489) of the patients, and 95% (471/489) were febrile upon admission (Table 1). The proportion of patients with a microscopy detected parasitaemia on day 3 (D3) post-DP treatment was highest in the Binh Phuoc site and increased progressively with time: 38, 41, 54, and 57% in 2012, 2013, 2014, and 2015, respectively (P < 0.001) (Fig. 3). In Ninh Thuan, the percentage remained low (<10%), and the differences were not significant (P = 0.29). In Gia Lai, patients were only recruited during 2011 and 2015 The D3 positivity rates were 0% in Iato versus 55% in Krong Pa (P < 0.001). The parasite clearance half-life in Binh Phuoc increased from 3.75 h in 2011 to 6.60 h in 2015 (P < 0.001) (Tables 2, 3, 4; Fig. 3). One early treatment failure occurred at Binh Phuoc (Table 2). This patient was febrile with a positive blood smear on day 3, but recovered fully by the end of treatment. Treatment failure rates were highest in Binh Phuoc, and increased significantly over time (P < 0.001) as follows: 0% in 2012 and 2013 and 7 and 26% in 2014 and 2015, respectively (Table 2). There were only two late treatment failures in Ninh Thuan (Table 3) and none in Gia Lai (Table 4).

**Table 1:** Patient characteristics at enrolment sites in Vietnam

Characteristic	Binh Phuoc (N = 253)	NinhThuan (N = 188)	Gia Lai (N = 48)
Age [years]–median (IQR)	24 (18.33)	16 (10.26)	27 (22.29)
Children 3–14 years old-n (%)	23/253 (9.1%)	89/188 (47.3%)	1/48 (2.1%)
Female gender–n (%)	39/253 (15%)	70/188 (37%)	3/48 (6%)
Temperature [°C]–median [IQR]	39.0 (38.3–39.5)	38.8 (38.0–39.5)	39.5 (39.0–40.0)
Parasitaemia [parasites/µL]–median (IQR)	50,240 (29485,97654)	70,964 (30144,135648)	39,935 (23454,86500)

**Table 2:** Annual clinical and parasite responses to dihydroartemisinin-piperaquine treatment of uncomplicated *Plasmodium falciparum* malaria in Binh Phuoc province, Vietnam

Characteristic	2012 (n = 50)	2013 (n = 66)	2014 (n = 65)	2015 (n = 72)	P value*
Fever clearance time [hours]					
Median (IQR) [hours]	24 (18.30)	18 (12.24)	24 (18.36)	24 (18.36)	<0.001
Parasite clearance time (PCT100)					
Median (IQR) [hours]	63 (48.84)	60 (36.90)	78 (36.168)	78 (60.96)	0.002
Proportion with PCT > 72 h	38% (19/50)	41% (29/66)	54% (35/65)	57% (41/72)	
Parasite clearance half- life PC_1/2_ [hours]					
Median (IQR)	3.75 (2.45;6.56)	4.11 (2.10;6.27)	5.80 (2.45;7.14)	6.60 (4.65;8.03)	<0.001
Geometric mean (95% CI)	3.94 (3.38;4.60)	3.67 (3.16;4.26)	4.29 (3.66;5.03)	5.52 (4.84;6.29)	
T_1/2_ > 5 h	20/50 (40%)	27/64 (42%)	36/64 (56%)	51/69 (74%)	
Treatment outcome (WHO)					
APCR#	47/50 (94%)	55/66 (83%)	43/65 (66%)	40/72 (56%)	
Early treatment failure	0/50 (0%)	0/66 (0%)	1/65 (2%)	0/72 (0%)	
Clinical treatment failure	0/50 (0%)	0/66 (0%)	1/65 (2%)	8/72 (11%)	
Parasitological treatment failure	2/50 (4%)	1/66 (2%)	5/65 (8%)	12/72 (17%)	–
Withdrawal	1/50 (2%)	4/66 (6%)	2/65 (3%)	3/72 (4%)	
Loss to follow-up	0/50 (0%)	6/66 (9%)	13/65 (20%)	9/72 (13%)	
Risk of failure [PCR—uncorrected]					
Uncorrected Kaplan–Meier estimate (ITT)	4%	2%	12%	30%	<0.001
Uncorrected proportion (per protocol)	2/49 (4%)	1/56 (2%)	7/50 (14%)	20/60 (33%)	<0.001
Risk of failure [PCR—corrected]					
Corrected Kaplan–Meier estimate (‘ITT’)	0%	0%	5%	22%	<0.001
Corrected Proportion (‘per protocol’)	0/47 (0%)	0/55 (0%)	3/46 (7%)	14/54 (26%)	<0.001
K13-propeller mutation	I543T:1/50 (2%) C580Y:5/50 (10%) P553L:6/50 (12%) Y493H:7/50 (14%) Total 19/50 (38%)	I543T:3/65 (4.62%) C580Y:3/65 (3.08%) P553L:4/65 (6.15%) Y493H:14/65 (21.54%) 24/65 (37%)	I543T:1/65 (1.54%) C580Y:25/65 (38.5%) P553L:2/65 (3.08%) Y493H:1/65 (1.54%) 29/65 (45%)	C580Y:48/66 (72.73%)	<0.001

* P values are based on linear trend tests

*APCR* adequate clinical and parasitological responses

**Table 3:** Annual clinical and parasite responses to dihydroartemisinin-piperaquine treatment of uncomplicated *Plasmodium falciparum* malaria in Ninh Thuan province, Vietnam

Characteristic	2013 (n = 77)	2014 (n = 56)	2015 (n = 55)	P value*
Parasite clearance time (PCT100)				
Median (IQR) [hours]	36 (24.42)	30 (24.36)	30 (24.36)	0.29
Proportion with PCT > 72 h	5% (4/77)	7% (4/56)	2% (1/55)	
Parasite clearance half- life T_1/2_ [hours]				
Median (IQR)	1.73 (1.38; 2.05)	1.78 (1.44; 2.36)	2.16 (1.75; 2.73)	0.29
Geometric mean (95% CI)	1.84 (1.66; 2.03)	1.98 (1.73; 2.27)	2.19 (2.01; 2.39)	
T_1/2_ > 5 h	5/76 (7%)	5/56 (9%)	1/54 (2%)	
Fever clearance time [hours]				
Median (IQR) [hours]	30 (24.54)	18 (12.30)	18 (12.24)	<0.001
Treatment outcome (WHO)				
APCR#	62/77 (81%)	45/56 (80%)	43/55 (78%)	–
Early treatment failure	0/77 (0%)	0/56 (0%)	0/55 (0%)	
Clinical treatment failure	3/77 (4%)	1/56 (2%)	0/55 (0%)	
Parasite treatment failure	5/77 (6%)	1/56 (2%)	2/55 (4%)	
Withdrawal	2/77 (3%)	0/56 (0%)	0/55 (0%)	
Loss to follow-up	5/77 (6%)	9/56 (16%)	10/55 (18%)	
Risk of failure [PCR-uncorrected]				
Uncorrected Kaplan–Meier estimate (ITT)	11%	4%	4%	0.046
Uncorrected proportion (per protocol)	8/70 (11%)	2/47 (4%)	2/45 (4%)	0.05
Risk of failure [PCR-corrected]				
Corrected Kaplan–Meier estimate (ITT)	0%	4%	0%	0.81
Corrected proportion (per protocol)	0/62 (0%)	2/47 (4%)	0/43 (0%)	0.80
K13-propeller mutation	I543T: 1/77 (1%) Y493H: 4/77 (5%) Total 5/77 (6%)	C580Y: 2/56 (3.5%) Y493H:2/56 (3.5%) 4/56 (7%)	C580Y: 2/55 (4%)	0.53

* P values are based on linear trend tests

*APCR* adequate clinical and parasitological responses

**Table 4:** Annual clinical and parasite responses to dihydroartemisinin-piperaquine treatment of uncomplicated *Plasmodium falciparum* malaria in Gia Lai province, Vietnam

Characteristic	2011 (n = 21)	2015 (n = 27)	P value*
Parasite clearance time (PCT100)			
Median (IQR) [hours]	36 (30,42)	96 (54, NA)	<0.001
Proportion with PCT > 72 h	0% (0/21)	55% (15/55)	
Parasite clearance half- life T_1/2_ [hours]			
Median (IQR)	1.97 (1.66, 2.59)	6.88 (2.84, 7.74)	<0.001
Geometric mean (95% CI)	2.19 (1.86, 2.59)	5.19 (4.07, 6.62)	
T_1/2_ > 5 h	0/21 (0%)	18/26 (69%)	
Fever clearance time [hours]			
Median (IQR) [hours]	30 (18.30)	36 (24.54)	0.001
Treatment outcome (WHO)			
APCR#	17/21 (81%)	24/27 (89%)	–
Early treatment failure	0/21 (0%)	0/27 (0%)	
Clinical treatment failure	0/21 (0%)	0/27 (0%)	
Parasitological treatment failure	0/21 (0%)	2/27 (7%)	
Withdrawal	2/21 (10%)	0/27 (0%)	
Loss to follow-up	2/21 (10%)	1/27 (4%)	
Risk of failure [PCR-uncorrected]			
Uncorrected Kaplan–Meier estimate (ITT)	0%	8%	0.22
Uncorrected proportion (per protocol)	0/17 (0%)	2/26 (8%)	0.22
Risk of failure [PCR-corrected]			
Corrected Kaplan–Meier estimate (ITT)	0%	0%	–
Corrected proportion (per protocol)	0/17 (0%)	0/24 (0%)	–
K13-propeller mutation	0/20 (0%)	C580Y:18/27 (67%) Y493H:2/27 (7%) Total 20/27 (74%)	<0.001

* P values are based on linear trend tests

*APCR* adequate clinical and parasitological responses

Samples for evaluation of mutations were available for 481 subjects. K13-propeller mutations (n = 151, 31%) were as follows: 109, C580Y; 24, Y493H; 10, P553L; 7, I543T and 1 V568G (Additional File 2). Baseline parasitaemia was associated with the K13-propeller mutation (*P* value = 0.37 [linear regression, adjusted for site]). The median (IQR) parasite clearance half-life in patients with a K13-propeller mutation was 6.59 h (5.14–7.42 h) compared with 2.05 h (1.66–2.90 h) in subjects without the mutation [P < 0.001 (linear regression, adjusted for site)]. Half-lives were longer in subjects with a C580Y mutation compared with those with Y493H mutations, although the difference was not statistically significant (median 6.70 and 5.42 h, P = 0.07). Among the 17 subjects with PCR-corrected treatment failure in Binh Phuoc, 15 had a K13-propeller mutation (all C580Y), one did not have the mutation, and one did not provide a sample.

**Fig. 4:**
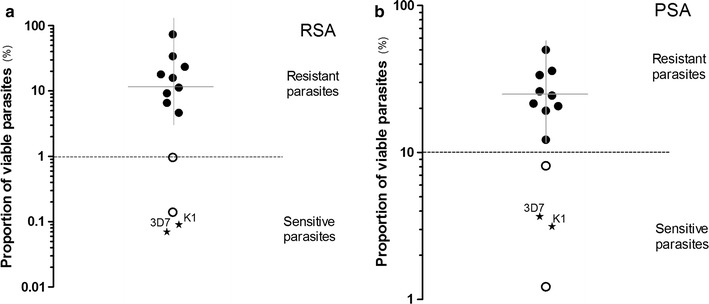
Ex vivo response to DHA and in vitro response to piperaquine of parasites collected from patients following dihydroartemisin–piperaquine treatment using ring stage assay (RSA) and piperaquine survival assay (PSA), respectively. *Black dots* represented for parasites from recrudescent cases, *white dots* represented for parasite from ACPR cases. The *Plasmodium falciparum* lines of 3D7 and K1 are controls

Ex vivo responses to DHA were measured in 64% (9/14) of *P. falciparum* isolates collected from recrudescent patients in 2015 by RSA and all were resistant to DHA (survival rate >1), with a median survival rate of 14.29% (IQR 4.76%, 23.51%). The 9 isolates were also tested in the PSA and all were resistant to piperaquine, with survival rate ≥10% (Fig. 4). The median survival rate after exposure to 200 nM of piperaquine was 20.72% (IQR 8.28%, 30.07%). The RSA and PSA were also carried out on isolates from two patients who experienced an ACPR response to DP.

**Fig. 5:**
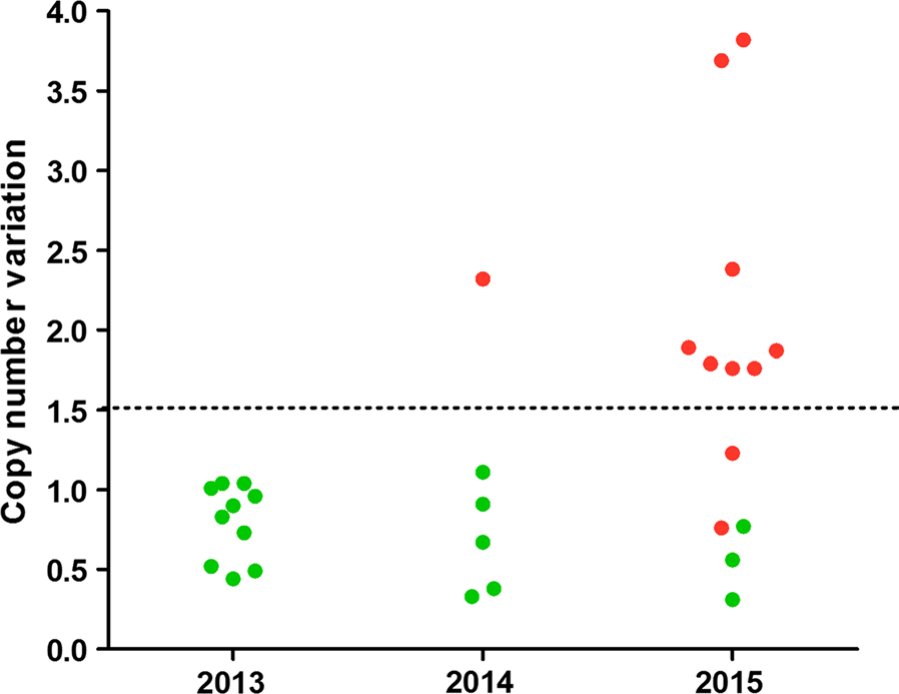
Copy variation number of plasmepsin 2 gene in the parasites collected from patients following dihydroartemisin–piperaquine treatment. *Green dots* represented to parasites form ACPR cases. *Red dots* represented to parasite form recrudescent cases

The exoE415G mutation, which has been reported associated with increased tolerance to piperaquine [12] were found in 93% (13/14) isolates collected from recrudescent patients in 2015. Due to limited DNA amount of the recrudescent samples, plasmepsin 2 amplification was measured in 71% (10/14) of the samples. Eight of them (80%) had multi-copies of this gene (Fig. 5).

## Discussion

The widespread use of artemisinin and its derivatives in Vietnam has been a major factor underlying the marked reduction in malaria mortality and morbidity in the country. Until recently the nationally recommended ACT, dihydroartemisinin-piperaquine, retained excellent efficacy throughout the country.

Since the first recognition of artemisinin resistance in Cambodia, studies have been conducted to assess whether resistance had emerged in or spread to Vietnam, and what impact that would have on treatment efficacy. In 2010–2011, a randomized controlled clinical trial of 166 patients with uncomplicated *P. falciparum* malaria in the Binh Phuoc area showed that the proportion of patients with parasite clearance times >72 h ranged from 22 to 25%, depending on the dose of artesunate given (2 or 4 mg/kg/day). The corresponding median parasite clearance half-lives (PC_1/2_) for were <5 h. Both indicated a high prevalence of slow parasite clearance. Although there were two ETFs (4%) and one late clinical failure (2%) in the lower dose artesunate (2 mg/kg/day) group, the PCR-corrected ACPR rates in the three groups were 94, 100, and 100% [14, 15]. This showed that artemisinin resistance was present in Vietnam but had not yet affected the efficacy of the first-line treatment.

This study reports longitudinal clinical, parasitological and molecular surveillance over a five year period from malaria endemic areas in the south of the country close to Eastern Cambodia. In Binh Phuoc province between 2012 and 2015 the proportion of patients with parasites still present on day 3 after starting DP treatment has progressively increased from 38 to 57%, with a corresponding reduction in cure rates from 0 to 26% (PCR corrected as per protocol). It provides unequivocal evidence of worsening artemisinin resistance and it shows that this has recently been associated with a rapid deterioration in the efficacy of DP. This strongly suggests that piperaquine resistance has arisen upon a background of artemisinin resistance reported recently from Cambodia [16]. The in vitro and molecular studies provided support for this; all patients who failed treatment were infected with K13 mutated parasites, and the parasites from recrudescent infections had median in vitro PSA survival rate of 39.2% compared with 0.17% in the patients who were cured with DP. Artemisinin-resistant K13 mutants with ex vivo PSA survival rates ≥10% were associated with a 32-fold higher risk of recrudescence [11]. It is notable that all 14 recrudescent patients in 2015–2016 were infected with the C580Y PfK13 genotype, which may indicate a recent hard selective sweep by a successful multi-drug resistant lineage.

While piperaquine was effective the DP combination provided high cure rates, but the reduced anti-malarial effect associated with artemisinin resistance places greater selection pressure on the partner drug, and when resistance to that occurs, the combination predictably fails. The detection of markers for piperaquine and DP treatment failure in the recrudescent patients also supported this. Among all the tested recrudescent parasites collected in 2015–2016, 93% (13/14) of them have exoE415G mutations and 80% (8/10) have multi copies of the plasmepsin 2 gene. It may indicate that resistance to piperaquine occurred. There has been insufficient attention to the protection of new anti-malarial medicines. Strategies such as deployment of multiple first line therapies are needed when effective alternatives are available [17]. In Vietnam, alternatives to DP are limited.

These findings present policymakers in Vietnam with a dilemma. The currently recommended second-line regimen combines quinine with doxycycline. But this regimen is not well tolerated and adherence to the required 7-day regimen is likely to be poor. The combination of artesunate with mefloquine may serve as an alternative. Vietnam used mefloquine both as a monotherapy and in an ACT (artesunate-mefloquine) since the 1990s. In 2005, foreign financial support ceased, and the NMCP of Vietnam, therefore, switched to locally produced DP as first-line treatment for falciparum malaria. In 2002–2003, a 2-day regimen of DHA-mefloquine (300 mg DHA at 0 h, then 300 mg DHA plus 750 mg mefloquine at 24 h) was evaluated at Binh Thuan and Binh Phuoc provinces and found to be 88.6% efficacious with a 42-day follow-up period. However, the limitation of the study was that it was not PCR corrected for differentiating rescrudescence from reinfection, and it is quite likely that a total dose of artesunate (12 mg/kg) plus mefloquine (25 mg/kg) given over 3 days would have been more effective. A 3-day artesunate-mefloquine regimen is presently being used in Cambodia in areas of DP treatment failure with cure rates of >99% (Chanaki et al. pers. comm.). A disadvantage of the mefloquine containing regimens is that they are not as well tolerated as DP [18–20]. Atovaquone–proguanil (ATQ–PG) was suggested as a non-artemisinin containing anti-malarial treatment option. ATQ–PG was assessed in a small trial conducted in 2003 by the NMCP of Vietnam (unpublished data) and the overall cure rate (CI) was 86% (76–98%) [21]. This is still lower than the 95% benchmark recommended by WHO. Recently, an in vitro study provided evidence that ATQ–PG may be a useful stopgap for DP failed cases [22], but a previous study has also shown that this combination remains susceptible to developing resistance [23]. Furthermore, because of the high costs of this drug ATQ–PG has not also been considered as an alternative treatment by the Vietnam NMCP.

Artemether-lumefantrine (AL, Coartem®) may be another treatment option, but no clinical trial to assess AL has been conducted in Vietnam over the last 20 years. A pilot trial was conducted in 2003 that resulted in 100% (45/45) APCR rate with 28 day follow-up (personal communication). However, Vietnam’s NMCP has not considered AL as viable treatment alternative to DP due to financial constraints. The future may belong to newer anti-malarials, such as cipargamin (KAE 609), KAF 156, and artefenomel (OZ 439), but these are in phase II of development [24] and will likely not reach the market until 2020.

## Conclusion

The authors conclude that there is no current medicine available to Vietnam’s national treatment guideline committee that meets the WHO criteria of being, “based on an average cure rate of >95%, as assessed in clinical trials.” There is an urgent requirement to evaluate alternative treatments with other available forms of ACT and to develop new anti-malarial drugs given standard courses of ACT are failing.

## Additional files

**Additional file 1.** The number of DP tablets administered to patients according to body weight.

**Additional file 2.** Proportion of K13 propeller mutations in the study population.
