# Supplementary material for: Rapid decline in the susceptibility of Plasmodium falciparum to dihydroartemisinin–piperaquine in the south of Vietnam
Source: Malar J. 2017 Jan 13;16:27. doi: 10.1186/s12936-017-1680-8 (PMC5237149; doi:10.1186/s12936-017-1680-8)
Supplement: Supplementary file 1 — Additional file 1. The number of DP tablets administered to patients according to body weight. [file 12936_2017_1680_MOESM1_ESM.pdf]

Additional File 1: The number of DP tablets administered to patients according to body weight.

Body weight	Day 0 (at 0 and 8h)	Day 1 (at 24h)	Day 2 (at 48h)
≥35 kg	2.0 - 2.0	2.0	2.0
25–34 kg	1.5 - 1.5	1.5	1.5
15–24 kg	1.0 - 1.0	1.0	1.0
<15 kg	0.5 - 0.5	0.5	0.5
