# Supplementary figures and images for: Rapid decline in the susceptibility of Plasmodium falciparum to dihydroartemisinin–piperaquine in the south of Vietnam
Source: Malar J. 2017 Jan 13;16:27. doi: 10.1186/s12936-017-1680-8 (PMC5237149; doi:10.1186/s12936-017-1680-8)

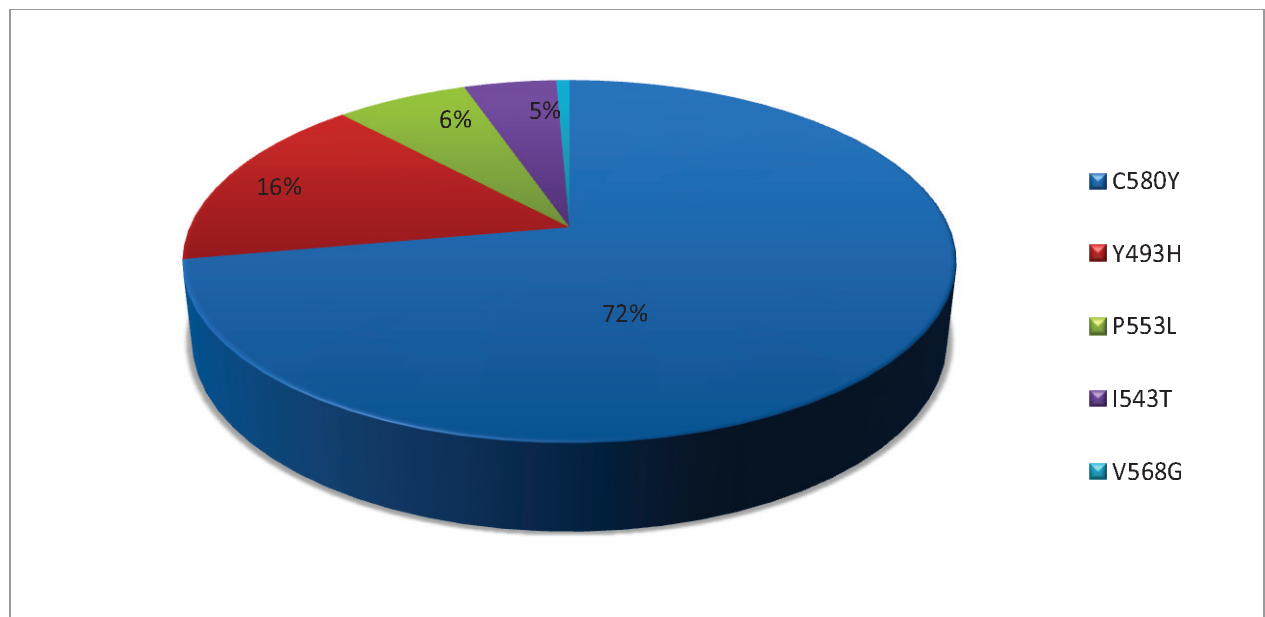

**Additional File 2: Proportion of K13 propeller mutations in the study population.**

Supplement: Supplementary file 2 — Additional file 2. Proportion of K13 propeller mutations in the study population. [file 12936_2017_1680_MOESM2_ESM.pdf]
